# Factors affecting mechanical complications of central venous access devices in children

**DOI:** 10.1007/s00383-022-05130-1

**Published:** 2022-05-05

**Authors:** Jessica J. Zhang, Ramesh M. Nataraja, Amiria Lynch, Richard Barnes, Peter Ferguson, Maurizio Pacilli

**Affiliations:** 1 Department of Paediatric Surgery, Monash Children’s Hospital, 246 Clayton Road, Clayton, VIC 3168 Australia; 2 Department of Paediatrics, School of Clinical Sciences, Monash University, Melbourne, Australia; 3 Department of Surgery, School of Clinical Sciences, Monash University, Melbourne, Australia; 4 Department of Anaesthesia, Monash Medical Centre, Melbourne, Australia

**Keywords:** Central venous access device, Implantable port device, Hickman line, Mechanical complication

## Abstract

**Purpose:**

Factors leading to mechanical complications following insertion of central venous access devices (CVADs) in children are poorly understood. We aimed to quantify the rates and elucidate the mechanisms of these complications.

**Methods:**

Retrospective (2016–2021) review of children (< 18 years old) receiving a CVAD. Data, reported as number of cases (%) and median (IQR), were analysed by Fisher’s exact test, chi-squared test and logistic regression analysis.

**Results:**

In total, 317 CVADs (245 children) were inserted. Median age was 5.0 (8.9) years, with 116 (47%) females. There were 226 (71%) implantable port devices and 91 (29%) Hickman lines. Overall, 54 (17%) lines had a mechanical complication after 0.4 (0.83) years from insertion: fracture 19 (6%), CVAD migration 14 (4.4%), occlusion 14 (4.4%), port displacement 6 (1.9%), and skin tethering to port device 1 (0.3%). Younger age and lower weight were associated with higher risk of complications (*p* < 0.0001). Hickman lines had a higher incidence of complications compared to implantable port devices [24/91 (26.3%) vs 30/226 (13.3%); *p* = 0.008].

**Conclusion:**

Mechanical complications occur in 17% of CVADs at a median of < 6 months after insertion. Risk factors include younger age and lower weight. Implantable port devices have a lower complications rate.

**Level of evidence:**

Level 4: case-series with no comparison group.

## Introduction

Central venous access devices (CVADs) play an important role in providing long-term venous access. They have multiple functions including haemodynamic monitoring, delivery of intravenous fluids, medications, blood products, and total parenteral nutrition (TPN) [1]. Complications of CVADs can present during the insertion (e.g., pneumothorax, haemorrhage, arterial puncture, cardiac rhythm dysfunction, etc.) or in the post-operative period (e.g., central line associated blood stream infection (CLABSI), fractures, etc.) [2, 3].

Complications occurring during the insertion are well documented [2, 4, 5]. Significant research has been undertaken to reduce the incidence of these complications by focusing on the use of an ultrasound (US)-guided technique. There is established high-level evidence supporting the use of US-guided venepuncture compared to either the open cut-down technique or the landmark-guided technique for CVADs insertion in children and adults [6–8]. US offers significant advantages for patient safety and procedural quality during the placement of CVADs and there are definite guidelines from over 30 international organizations/societies endorsing its primary use regardless of the anatomical site [9–12]. Similarly, CLABSIs, which account for the majority of post-operative complications, are the focus of a significant amount of research. Different interventions, including the use of chlorhexidine-impregnated dressings and intraluminal lock solutions, such as low molecular weight heparin, taurolidine or ethylenediaminetetraacetic acid, have been trialled to reduce CLABSIs [1].

However, there is little published data on the incidence of mechanical complications occurring after the insertion, such as CVAD fracture, occlusion and dislodgment. Furthermore, the factors leading to these complications are poorly investigated in children. A systematic review of post-operative complications in children conducted by Ullman et al. in 2015 showed that 25% of CVADs fail before therapy is completed, and a review of 93 randomised control trials and 22 registries on paediatric vascular access devices found that mechanical complications are poorly investigated despite contributing to up to 25% of CVAD failures [3, 13]. Therefore, further data are required to determine the mechanisms and risk factors behind mechanical complications of CVADs after insertion.

Our aim was to quantify the rates and elucidate the mechanisms of these complications.

## Methods

### Participants

Paediatric patients (< 18 years old) who underwent CVAD insertion or revision at a tertiary paediatric surgery institution between July 2016 and March 2021 were identified from a prospectively kept database and reviewed.

### Operative technique

All children underwent an US-guided insertion of the CVAD using a modified Seldinger technique (catheter inserted over peel-away sheath). The right internal jugular vein (IJV) was the preferred site; if this was not suitable due to thrombosis from previous cannulations, the left IJV and then the subclavian veins were utilised. Vein patency was assessed with US in all children before attempting venepuncture. The external diameter of the catheter was determined to maintain a catheter-to-vessel ratio less than 45% [14]. In all patients silicone lines were utilised; Bard implantable port devices and Hickman lines (Bard Access Systems, Inc., USA) and AngioDynamics implantable port devices (AngioDynamics, Inc., USA). Implantable port devices were positioned over the lateral chest wall and secured to the fascia using sutures including polypropylene, polyglactin 910, polydioxanone, and nylon.

Hickman lines were secured to the skin of the lateral chest wall using a nonabsorbable (polypropylene or nylon) suture and a subcutaneous anchoring device (SecurAcath^®^, Interrad Medical, USA) in the last 25 patients. Hickman lines, and implantable port devices with access needle in place, were regularly dressed with 3 M^™^ Tegaderm^™^ I.V. Advanced Securement Dressing (3 M Australia Pty Limited).

The position of the CVAD tip at the superior vena cava/right atrial junction was confirmed by fluoroscopy in all patients. All lines were aspirated and flushed at the end of the procedure and locked with 2.5 mL of heparinised saline 50 UI/5 mL. Lines were also locked with heparinised saline whilst not in use after insertion.

Patients’ files were reviewed for demographic data including: gender, age and weight at insertion, and diagnosis. Operative reports were reviewed to determine indications for CVAD insertion, site of insertion, proceduralist level, intra-operative conversion to a secondary site, CVAD details (including type, diameter, number of lumens), type of anchoring suture, perioperative complications and indication for revision.

Complication rates are expressed as frequencies, percentages and relative incidence (per 1000 catheter days). Failures (and other complications) per 1000 catheter days were calculated using the formula:

$$\mathrm{Failures\, per \,}1000\, \text{catheter-days}=\left(\frac{1000}{\sum \left(\mathrm{Catheter\, duration\, in\, days}\right)}\right)\times \mathrm{No}.\,\mathrm{ of\, failures}.$$

### Management of post-operative complications

#### Central line associated blood stream infection (CLABSI)

CLABSIs were diagnosed in the presence of the following three criteria:

1. Clinical signs of infection (e.g., fever, rigors, altered mental status, or hypotension);
2. No alternate source of bloodstream infection;
3. Positive blood culture from a peripheral vein.

In confirmed or suspected CLABSI, our approach is initially conservative, with attempts at preservation of the CVAD, but it is guided by patient’s specific considerations (e.g., presence of neutropenia, or residual duration of therapy). Vancomycin is used at our institution for the empiric treatment of CLABSIs as coagulase-negative *Staphylococci* are the most common causative microorganisms. Additional coverage for Gram-negative bacilli, with a fourth-generation cephalosporin, is considered in patients with severe illness, sepsis, a known focus of Gram-negative bacterial infection, or immunocompromised states, including neutropenia or malignancy. The duration of therapy for CLABSI is dependent on the organism suspected or isolated as well as evidence for endovascular sequelae such as venous thrombosis and CVAD occlusion. Treatment of an uncomplicated CLABSI due to Enterococcus or Gram-negative bacilli ranges from 7 to 14 days, while *S. aureus* may require up to 4 weeks.

#### Fracture

Hickman lines that have an external fracture more than 3 cm from the skin entry site, with no evidence of local or systemic infection, undergo repair before being considered for replacement; we use the appropriately sized external catheter repair segment kit (Bard Access Systems, Inc., USA) with a sterile technique according to the manufacture recommendations. Fractured implantable port devices are removed and replaced with new devices.

#### Occlusions

There are essentially three causes of occlusion:

1. Mechanical (e.g., clamp or kink in the intravenous tubing, malpositioned implanted port device needle, kinked line under the dressing, catheter tip malposition, etc.) that require specific management;
2. Chemical: resulting from the mixing of incompatible medications, solutions or build-up of debris within the CVAD lumen;
3. Thrombotic: resulting from an accumulation of fibrin or blood clot within the lumen of the CVAD.

Our protocol for chemical and thrombotic occlusions includes first an attempt with a high-pressure flush using diluted heparinised saline (10 units per mL) in a 2 mL syringe followed by an attempt to flush the line with tissue plasminogen activator (0.5 mg in 2 mL for patients < 10 kg and 2 mg in 2 mL for patients > 10 kg).

### Statistical analysis

Statistical analysis was performed using GraphPad Prism version 9.2.0 (GraphPad Software, San Diego, California USA) and MedCalc 20.014 (MedCalc Software Ltd). Data were reported as number of cases (%) and median (IQR) and analysed by Fisher’s exact test, chi‐squared test and logistic regression analysis. *p* value of < 0.05 was considered significant.

## Results

### Participants

During the study period there were 245 paediatric patients identified with a total of 317 CVADs being inserted. Seven patients who had completed their treatment required a new catheter for recurrence of their disease. In total, 54/317 (17%) had a mechanical complication which required a new catheter. The median age was 5.0 years (IQR 8.9). There were 116 (47%) females and 129 (53%) males.

Children who underwent insertion of a Hickman line were significantly younger than children receiving an implantable port device: 1.8 (6.2) vs 6.9 (15.2), *p* < 0.0001.

The underlying diagnoses and indications for CVADs insertion are reported in Table 1.

**Table 1:** Diagnosis and indication for central venous catheter insertion

Diagnosis	Number (%)
Leukaemia	101 (32)
Solid tumours	70 (22)
Lymphoma	55 (17)
Intestinal failure	34 (11)
Brain tumours	24 (8)
Haematological	14 (4)
Renal pathology	11 (4)
Long-term antibiotic therapy	8 (3)

Consultant surgeons inserted 107/317 (34%) CVADs and trainees 210/317 (66%). There were 226/317 (71%) implantable port devices and 91/317 (29%) Hickman lines. There were 232/317 (73%) single-lumen and 85/317 (27%) double-lumen CVADs. Insertion sites were right IJV in 270/317 CVADs (85%), left IJV in 44/317 (14%), and right subclavian in 3/317 (1%). There were no intra-operative complications. Median follow-up was 275 (652.5) days.

### Infective complications

CLABSI occurred in 34 (11%) CVADs at a median time of 0.42 (0.49) years equivalent to 0.37 × 1000 catheter days. Of these 20/245 (59%) underwent insertion of a new line after removal of the infected catheter. The remaining 14 (41%) CLABSIs were treated with antibiotic therapy without need for removing the catheter.

### Mechanical complications

Overall, 54 (17%) CVADs had a mechanical complication at a median of 0.4 (0.83) years from insertion, equivalent to 0.6 × 1000 catheter days. Forty-five (83%) required a new catheter; 16 (24%) oncological patients were treated with a percutaneous inserted central catheter (PICC) for the remaining duration of their therapy.

Those with complications were significantly younger [2.7 (5.5) vs 5.5 (9.2) years, (*p* < 0.0001)] and had lower weight [14.5 (15.6) vs 20 (28.2) kg, *p* = 0.0001] than those without complications. These findings were confirmed in a multiple logistic regression analysis with younger age and lower weight independently associated with higher risk of complications: age OR 0.76 (95% CI 0.66–0.87), *p* = 0.0001; weight OR 1.03 (1.01–1.06), *p* = 0.005.

A breakdown of mechanical complications is shown in Table 2.

**Table 2:** Mechanical complications

Complication	Implantable port device (*N* = 226)	Hickman (*N* = 91)	*p* value
Fracture	8 (3.5%)	11 (12%)^a^	**0.007**
Occlusion	9 (4%)	5 (5.5%)	0.5
Catheter dislodgment	6 (2.7%)	8 (8.8%)	**0.01**
Port dislodgment	6 (2.7%)	N/A	
Skin tethering to port device	1 (0.4%)	N/A	
Total	30 (13.3%)	24 (26.3%)	**0.008**

Statistically significant* p* values are in bold

^a^All fractures were external, distal to the tunnelled cuff

Hickman lines had a significantly overall higher mechanical complication rate compared to implantable port devices: 24/91(26%) vs. 30/226(13%), *p* = 0.008. Specifically, fractures of the external component of the Hickman line occurred in 11/91 (12%) patients; 3 of these fractures were suitable for repair and 8 required removal and re-insertion.

Catheter tip dislodgment was also more common in patients with Hickman lines (*p* = 0.01).

Mechanical complication rate was similar between lines inserted by consultant surgeons and trainees: 18/107 (17%) vs. 36/210 (17%), *p* = 0.9. There was no difference between lines inserted in the right or left IJV: 45/270 (17%) vs 9/44 (20%); *p* = 0.5.

Mechanical complications occurred in 42/232 (18%) single-lumen CVADs and in 11/85 (13%) double-lumen CVADs (*p* = 0.3). Similarly, the diameter of the catheter did not affect the rate of complications in the logistic regression analysis (*p* = 0.6).

Mechanical complications and anchoring methods are summarised in Table 3; the type of anchoring suture used was not available in 48 CVADs but none of these patients experienced dislodgment.

**Table 3:** Dislodgment by anchoring suture/device

Anchoring material	Total number (*N*)	Dislodgment (%)
Polypropylene	139	11 (8)
Polyglactin 910	87	6 (7)
Subcutaneous anchoring device	25	2 (8)
Polydioxanone	14	1 (7)
Nylon	4	0

The suturing material used to anchor the CVAD did not affect the incidence of dislodgment (*p* = 0.9).

## Discussion

Our study has identified a 17% overall incidence of post-insertion mechanical complications in 317 CVADs over a 5-year period. This detected incidence falls within the previously reported range of published data (9–60.3%) [4, 15–23]. Mechanical complications following CVAD insertion and potential causative factors are poorly investigated in children [13]. In our study mechanical complications occurred more commonly than CLABSIs. Noticeably, the reduction of CLABSIs is a priority in all health organisations around the world. Our institution has put in place many interventions, such as the use of chlorhexidine-impregnated dressings, catheter lock solutions and education of health care workers to reduce the incidence of CLABSIs.

Overall, we found that mechanical complications were more common in children with Hickman lines than implantable port devices. Similarly, a previous study of 499 children showed that the type of CVAD is the most significant modifiable risk factor for failure, with implantable port devices having the lowest rate of mechanical complication. [19]

Specifically, fracture of the external component of the Hickman lines occurred in 12% of our patients. CVAD fracture is believed to occur due to shearing forces to the catheter, high intra-catheter pressure during infusion, increased right ventricular pressure, and growth or movement of the patient [24–26]. Indeed, Hickman lines in young children are likely to be subjected to accidental rotational and tugging forces that might damage the line making it prone to fractures. Nonetheless, we found that fractures also happened in 3.5% of implantable port devices. At present, Hickman lines and implantable port devices used at our institution are made of silicone and are not power injectable. New generation lines, made of polyurethane are being implemented at our institution. Polyurethane allows the line to expand and contract during a power injection: to put this into perspective, a 10 mL syringe can exert a pressure up to 16 psi and power injectable lines can withstand pressures around 300 psi compared to silicone catheters which will rupture at pressures in excess of 25 psi. Due to the retrospective nature of the study, we were not able to identify the causes of catheter fracture, but we observed that this often happens in blocked catheters while significant force is used to flush or aspirate. Large prospective studies are required to confirm if polyurethane catheters will reduce the incidence of fractures in CVADs in children.

Interestingly, we found that younger age and lower weight were independently associated with a higher incidence of mechanical complications. This is consistent with previously published studies, especially with a younger age [19, 27, 28]. However, data on the association between a child’s weight and CVAD complications are lacking. Our study adds to the literature by showing a trend that mechanical complication rate appears to be higher in children with lower weight. This is consistent with a retrospective cohort study that showed that peripherally inserted CVAD failure is significantly associated with lower body weight at insertion [27].

Another complication in our series was occlusion (9.5% of patients), with a similar rate in Hickman lines and implanted port devices. Previous studies showed that complete or partial catheter occlusion affects 16–58% of patients after two years of CVAD placement [29]. This discrepancy is potentially related to the relatively short median follow-up in our study of 275 days. The main cause of catheter occlusion is the development of a fibrin sheath around the tip of the catheter and/or intraluminal thrombi which increase with time [30]. The fibrin sheath may lead to the inability to aspirate blood in otherwise asymptomatic patients, which is caused by a one-way valve mechanism created at the catheter opening [29]. Unfortunately, due to the retrospective nature of our study, we were not able to determine the role of the fibrin sheath in the blocked catheters which is a focus for future research in our department. The fibrin sheaths can be managed pharmacologically, with local or systemic thrombolytic agents, or mechanically, with transfemoral percutaneous intravascular stripping techniques or sheath disruption via balloon catheter [30]. A meta-analysis of eight studies (1428 patients) did not find benefits in terms of catheter blockage in patients receiving anticoagulation prophylaxis [31]. In contrast, the Cochrane Systematic Review by Akl et al. and Kahale et al. showed that the use of low-molecular weight heparin was associated with a statistically significant reduction in catheter thrombosis [32, 33]. There is emerging evidence that the use of taurolidine and ethylenediaminetetraacetic acid as catheter locking solutions may reduce the incidence of both CLABSIs and occlusions in children when compared to low-molecular weight heparin [34, 35]. In our current institutional practice, each CVADs lumen is locked after use with 2.4 mL of heparinised saline 50 UI/ml. However, we have only recently started using taurolidine locks in children with Hickman lines on home parenteral nutrition and so this is not captured in our dataset.

There was no association between the mechanical complication rates of CVADs inserted by either consultant surgeons or trainees. Rey et al. have suggested that mechanical complications at the time of insertion are more common in procedures performed by trainees. However, the mechanical complications in our study were after the perioperative timeframe, and therefore it is unlikely that the level of expertise of the operator will affect the incidence of these complications.

In our study, the mechanical complication incidence was not associated with the number of lumens or size of the catheter. Clinical practice guidelines recommend the use of the minimum number of lumens required to provide therapy, as studies have found an increased risk of infection, occlusion, and thrombosis in multi-lumen vascular access devices [36]. Our practice includes choosing the external diameter of the catheter to maintain a catheter-to-vessel ratio less than 45%. This is based on evidence that a higher ratio will cause a 13-fold increase in the catheter-related thrombosis [14].

There was no association between the mechanical complications and anchoring methods summarised in Table 3. The suturing material used to anchor the CVAD did not affect the incidence of dislodgment (*p* = 0.9). We are not aware of any study that has investigated the incidence of implanted port devices dislodgment using different sutures. Recently, subcutaneous anchoring devices for CVADs with external component (e.g., Hickman lines and PICC lines) have become available on the market and appear to be well tolerated and highly effective in preventing dislodgment, both in cuffed and non-cuffed catheters [37]. We have only used one of these devices (SecurAcath®, Interrad Medical, USA) in 25 Hickman lines, 2 (8%) of which got dislodged, so our experience is limited to draw any conclusion. We also dress our Hickman lines and accessed port devices with 3 M^™^ Tegaderm^™^ I.V. Advanced Securement Dressing (3 M Australia Pty Limited); however, due to the retrospective nature of our study, we were unable to investigate the role of this dressing in reducing the risk of dislodgement.

We found no difference between lines inserted in the right or left IJV which is consistent with the existing literature [25, 38, 39]. It has been suggested that the amount of contact by neighbouring anatomical structures and frequency of handling may influence this incidence of mechanical complications [25]. However, the right and left sides of the neck are not significantly different, which may explain the similar complication rate between the two sites.

Our finding that skin tethering to port device is rare is consistent with existing studies reporting this complication in 1.87–3.5% of CVADs [40–42].

Our study was limited by its retrospective, single-centre design, therefore, prospective multi-institutional studies are required to confirm our findings.

In conclusion, our study has shown that mechanical complications of CVADs are a relatively common occurrence in the paediatric population, with 17% of CVADs affected. Younger age, lower weight and Hickman line are all factors predisposing to CVAD failure. Therefore, implantable port devices might be more suitable for younger children whenever possible.

Fracture and occlusion are the most common complications; they might be reduced with the use of new generation polyurethane catheters and the adoption of locking solutions.

## References

[1] Gorski LA, Hadaway L, Hagle ME (2021). Infusion therapy standards of practice, 8th edition. J Infus Nurs.

[2] Rey C, Alvarez F, De La Rua V (2009). Mechanical complications during central venous cannulations in pediatric patients. Intensive Care Med.

[3] Ullman AJ, Marsh N, Mihala G, Cooke M, Rickard CM (2015). Complications of central venous access devices: a systematic review. Pediatrics.

[4] Fratino G, Castagnola E, Carlini C (2004). A single institution observational study of early mechanical complications in central venous catheters (valved and open-ended) in children with cancer. Pediatr Surg Int.

[5] Shilati FM, Raval MV, Lautz TB (2021). Technical factors and outcomes in pediatric central venous port placement. J Pediatr Surg.

[6] Brass P, Hellmich M, Kolodziej L (2015). Ultrasound guidance versus anatomical landmarks for internal jugular vein catheterization. Cochrane Database Syst Rev.

[7] Willetts IE, Ayodeji M, Ramsden WH (2000). Venous patency after open central-venous cannulation. Pediatr Surg Int.

[8] Wragg RC, Blundell S, Bader M (2014). Patency of neck veins following ultrasound-guided percutaneous Hickman line insertion. Pediatr Surg Int.

[9] Lamperti M, Biasucci DG, Disma N (2020). European Society of Anaesthesiology guidelines on peri-operative use of ultrasound-guided for vascular access (PERSEUS vascular access). Eur J Anaesthesiol.

[10] Lamperti M, Bodenham AR, Pittiruti M (2012). International evidence-based recommendations on ultrasound-guided vascular access. Intensive Care Med.

[11] Leibowitz A, Oren-Grinberg A, Matyal R (2020). Ultrasound guidance for central venous access: current evidence and clinical recommendations. J Intensive Care Med.

[12] Spencer TR, Pittiruti M (2019). Rapid Central Vein Assessment (RaCeVA): a systematic, standardized approach for ultrasound assessment before central venous catheterization. J Vasc Access.

[13] Schults JA, Rickard CM, Kleidon T (2019). Building a global, pediatric vascular access registry: a scoping review of trial outcomes and quality indicators to inform evidence-based practice. Worldviews Evid Based Nurs.

[14] Spencer TR, Mahoney KJ (2017). Reducing catheter-related thrombosis using a risk reduction tool centered on catheter to vessel ratio. J Thromb Thrombolysis.

[15] Fratino G, Mazzola C, Buffa P (2001). Mechanical complications related to indwelling central venous catheter in pediatric hematology/oncology patients. Pediatr Hematol Oncol.

[16] Fratino G, Molinari AC, Mazzola C (2002). Prospective study of indwelling central venous catheter-related complications in children with broviac or clampless valved catheters. J Pediatr Hematol Oncol.

[17] Fratino G, Molinari AC, Parodi S (2005). Central venous catheter-related complications in children with oncological/hematological diseases: an observational study of 418 devices. Ann Oncol.

[18] Gambarara M, Ferretti F, Papadatou B (2001). Central vein catheter-related complications associated with home parenteral nutrition in children: experience in 41 patients. Nutrition.

[19] Bough G, Lambert NJ, Djendov F (2021). Unexpected tunnelled central venous access demise: a single institutional study from the UK. Pediatr Surg Int.

[20] Cesaro S, Cavaliere M, Pegoraro A (2016). A comprehensive approach to the prevention of central venous catheter complications: results of 10-year prospective surveillance in pediatric hematology-oncology patients. Ann Hematol.

[21] Hussain S, Gomez MM, Wludyka P (2007). Survival times and complications of catheters used for outpatient parenteral antibiotic therapy in children. Clin Pediatr (Phila).

[22] Ruebner R, Keren R, Coffin S (2006). Complications of central venous catheters used for the treatment of acute hematogenous osteomyelitis. Pediatrics.

[23] Cesaro S, Corro R, Pelosin A (2004). A prospective survey on incidence and outcome of Broviac/Hickman catheter-related complications in pediatric patients affected by hematological and oncological diseases. Ann Hematol.

[24] Biswas S, McNerney P (2015). Ventricular tachycardia from a central line fracture fragment embolus: a rare complication of a commonly used procedure-a case report and review of the relevant literature. Case Rep Crit Care.

[25] Duesing LA, Fawley JA, Wagner AJ (2016). Central venous access in the pediatric population with emphasis on complications and prevention strategies. Nutr Clin Pract.

[26] Ares G, Hunter CJ (2017). Central venous access in children: indications, devices, and risks. Curr Opin Pediatr.

[27] Kleidon TM, Rickard CM, Schults JA (2020). Development of a paediatric central venous access device database: a retrospective cohort study of practice evolution and risk factors for device failure. J Paediatr Child Health.

[28] Ordonez J, Del Canizo A, Belendez C (2021). Complications of central venous access devices in patients with sickle cell disease and thalassemia major. J Pediatr Hematol Oncol.

[29] Lichtenstein T, Mammadov K, Rau K (2021). Long-term follow-up and clinical relevance of incidental findings of fibrin sheath and thrombosis on computed tomography scans of cancer patients with port catheters. Ther Clin Risk Manag.

[30] Baskin JL, Pui CH, Reiss U (2009). Management of occlusion and thrombosis associated with long-term indwelling central venous catheters. Lancet.

[31] Chaukiyal P, Nautiyal A, Radhakrishnan S (2008). Thromboprophylaxis in cancer patients with central venous catheters. A systematic review and meta-analysis. Thromb Haemost.

[32] Akl EA, Vasireddi SR, Gunukula S (2011). Anticoagulation for patients with cancer and central venous catheters. Cochrane Database Syst Rev.

[33] Kahale LA, Tsolakian IG, Hakoum MB (2018). (2018) Anticoagulation for people with cancer and central venous catheters. Cochrane Database Syst Rev.

[34] Quirt J, Belza C, Pai N (2021). Reduction of central line-associated bloodstream infections and line occlusions in pediatric intestinal failure patients receiving long-term parenteral nutrition using an alternative locking solution, 4% tetrasodium ethylenediaminetetraacetic acid. J Parenter Enteral Nutr.

[35] Sun Y, Wan G, Liang L (2020). Taurolidine lock solution for catheter-related bloodstream infections in pediatric patients: a meta-analysis. PLoS ONE.

[36] Paterson RS, Chopra V, Brown E (2020). Selection and insertion of vascular access devices in pediatrics: a systematic review. Pediatrics.

[37] Crocoli A, Martucci C, Sidro L (2021). Safety and effectiveness of subcutaneously anchored securement for tunneled central catheters in oncological pediatric patients: a retrospective study. J Vasc Access.

[38] Unal AE, Bayar S, Arat M (2003). Malpositioning of Hickman catheters, left versus right sided attempts. Transfus Apher Sci.

[39] Camkiran Firat A, Zeyneloglu P, Ozkan M (2016). A randomized controlled comparison of the internal jugular vein and the subclavian vein as access sites for central venous catheterization in pediatric cardiac surgery. Pediatr Crit Care Med.

[40] Bass J, Halton JM (2009). Skin erosion over totally implanted vascular access devices in children. Semin Pediatr Surg.

[41] McMahon C, Smith J, Khair K (2000). Central venous access devices in children with congenital coagulation disorders: complications and long-term outcome. Br J Haematol.

[42] De Backer A, Vanhulle A, Otten J (1993). Totally implantable central venous access devices in pediatric oncology–our experience in 46 patients. Eur J Pediatr Surg.

